# A model of population dynamics of TB in a prison system and application to South Africa

**DOI:** 10.1186/s13104-017-2968-z

**Published:** 2017-11-29

**Authors:** Peter Witbooi, Sibaliwe Maku Vyambwera

**Affiliations:** Department of Mathematics and Applied Mathematics, University of the Western Cape, Private Bag X17, Bellville, 7535 Republic of South Africa

**Keywords:** Prison TB model, Inflow of infecteds, Removal rate

## Abstract

**Background:**

Tuberculosis (TB) continues to spread in South African prisons in particular, as prisons are over-capacitated and have poor ventilation. The awaiting trial detainees are not screened on admission and are at high risk of getting infected with TB.

**Results:**

We propose a compartmental model to describe the population dynamics of TB disease in prisons. Our model considers the inflow of susceptible, exposed and TB infectives into the prison population. Removal of individuals out of the prison population can be either by death or by being released from prison, as compared to a general population in which removal is only by death. We describe conditions, including non-inflow of infectives into the prison, which will ensure that TB can be eradicated from the prison population. The model is calibrated for the South African prison system, by using data in existing literature. The model can be used to make quantitative projections of TB prevalence and to measure the effect of interventions. Illustrative simulations in this regard are presented. The model can be used for other prison populations too, if data is available to calculate the model parameters.

**Conclusions:**

Various simulations generated with our model serve to illustrate how it can be utilized in making future projections of the levels of prevalence of TB, and to quantify the effect of interventions such as screening, treatment or reduction of transmission parameter values through improved living conditions for inmates. This makes it particularly useful as there are various targets set by the World Health Organization and by governments, for reduction of TB prevalence and ultimately its eradication. Towards eradication of TB from a prison system, the theorem on global stability of the disease-free state is a useful indicator.

## Background

The World Health Organization (WHO) has recently launched the *End TB Strategy* program with the aim to reduce the number of deaths due to tuberculosis (TB) and the TB incidence rate by 95% and 90% in 2030, respectively. Their focus will be the most vulnerable who are infected by TB such as the poor, refugees, HIV-infected people and prisoners. The three main pillars of the program are: integrated patient centered TB care and prevention, bold policies and supportive systems and intensified research and innovation [20].

Prisons have been recognized internationally as institutions with very high tuberculosis burden as compared to a general population [13]. South African prisons are well known as being overcrowded. In 2015, 61 of the 90 centres in South Africa were inspected and it was found that their occupancy were more than 100% [14, p. 52]. The National Strategic Plan 2012–2016 [17, 18] of the Department of Health is aimed at reduction of TB infection. It has prioritized TB screening in prison and mines in view of overcrowding in these premises. The pipeline report for 2013 [18] points out factors that aggravates TB transmission. The transmission of TB in a prison is driven by the amount of air shared between inmates, the number of inmates per cell, the length of the lock-up time, how much fresh airflow is used and the presence of infectious inmates in the same enclosure with susceptible inmates. Awaiting trial inmates are being kept in a very intensely crowded environment. So for instance one could have as many as 86 inmates in a facility which was designed for 20, sharing a single toilet [19]. The Department of Correctional Services admits that overcrowding is a major problem in prisons. In Robertson et al. [7] a mathematical model is developed to explore the incarceration conditions and TB control measures. In this paper we model the population dynamics of the TB disease in a prison population with special emphasis on the South African prison system. The focus in [7] is on the effective contact rate, which in the present paper is denoted by $c_1$. In this work we quantify the broader effect of $c_1$ on the prevalence of the TB infection. In the literature already, the paper [10] considers a mathematical model for assessing the population dynamics of HIV and HCV co-existence within correctional facilities.

The current paper presents a deterministic compartmental model ordinary differential equations. A prison model must consider the inflow of infected people into the system. The removal rate in the case of a prison population is completely different from the case of a general population. For a prison population, individuals are removed not only through death, but also by being released. We give detail on the general method of calculating the removal rates from the system. We make specific calculations in the South African context, and we determine other parameters and input data for the model. Our Theorem 1 determines threshold conditions that will ensure the eradication of TB disease from the prison. Finally, we illustrate the utility of the model and of the theorem through simulations.

## Methods

### The model

We introduce a deterministic compartmental model based on the papers [4] of Buonomo and Lacitignola and [12] of Ssematimba et al., the latter two papers being on tuberculosis in concentration camps. This type of very dense population necessarily has a very high contact rate between the individuals, in particular healthy susceptible people are in very close and frequent contact with people having infectious active TB. Due to the similarities between concentration camps and prisons such as overcrowding, the amount of air shared between the individuals etc., we consider this model, modified to accommodate inflow of infecteds, to be applicable to prison populations.

The prison population consists of sentenced prisoners together with awaiting trial detainees, and the size of the population at time *t* is denoted by *N*(*t*). We divide the population into four compartments namely, susceptible individuals *S*(*t*), individuals with active TB who are not infectious, *E*(*t*), individuals infected with active TB who are infectious *I*(*t*), and the class of individuals under treatment *T*(*t*) [and often these variables will be written without stressing the dependence on the time variable (t)]. Due to the classes used, the model is referred to as being of *SEIT* type. We modify the model of Buonomo and Lacitignola [4] by allowing for the inflow of exposed individuals and infectious individuals into the prison population.

It is important to note that in general populations, removal of individuals out of the system is only by death. In this model, removal is by death or by discharge from prison, and the discharge is the dominant factor. This rate of removal is denoted by $\mu$. In the classes *S*, *E* and *T* the probability of an individual being removed from the class is denoted by $\mu$, and will be referred to as the *removal rate*. For the class *I*, mortality due to TB-disease amplifies the removal rate by an additional increment *d*, which will be referred to as the disease-induced mortality rate. The total inflow into the population is assumed to be at a rate $A_0$. We find it useful to express $A_0$ in the form $A_0=\mu A$ for some constant positive number *A* and with $\mu$ being the removal rate. The number *A* will be seen to be the upper limit of *N*(*t*). We assume that there are non-negative numbers $f_{S},\, f_{E} ~\mathrm{and} ~f_{I}$ such that the inflow into the classes of (respectively) susceptible, exposed and infectious happen at the rates $f_{S}\mu A, \, f_{E}\mu A ~\mathrm{and} ~f_{I}\mu A$, respectively.

Susceptible individuals get infected with active TB at a rate $c_{1} SI$, where $c_{1}$ is the effective contact rate between the infectious and susceptible individuals. Individuals leave the exposed class *E*(*t*) for the infectious class *I*(*t*) at a rate $k{E} + c_{3}EI$, where $c_{3}$ is the effective contact rate between the exposed and infectious individuals. Successfully treated individuals who were infectious move to exposed class at a rate $c_{2}TI$. Exposed and infectious individuals move into treatment class *T*(*t*) at a rate $r_{1}E$ and $r_{2}I$ respectively.

1 $$\begin{aligned} {\mathop {S}\limits ^{.}}= \,& {} f_{S}\mu {A}-c_{1}SI-\mu {S},\nonumber \\ {\mathop {E}\limits ^{.}}= \, & {} f_{E}\mu {A}+c_{1}SI+c_{2}TI-c_{3}EI-(\mu +r_{1}+k)E,\nonumber \\ {\mathop {I}\limits ^{.}}= & \, {} f_{I}\mu {A}+kE-(\mu +r_{2}+d)I+c_{3}EI,\nonumber \\ {\mathop {T}\limits ^{.}}= \, & {} r_{1}E+r_{2}I-c_{2}TI-\mu {T}. \end{aligned}$$

If $f_{E}+f_{I}>0,$ then our model system (1) does not have a disease free equilibrium due to the fact that there is an inflow of infectives into the prison population. Thus it is clear that TB in prison cannot be eliminated as long as the wider population has individuals with active TB that go to prison.

We first study the model without the inflow of infectives. If $f_{E}=0 ~\mathrm{and}~ f_{I}=0,$ then the model given by the system (1) has a unique feasible disease free equilibrium given by

$$\begin{aligned} P_{0}=(S_{0},E_{0},I_{0},T_{0})=(A,0,0,0). \end{aligned}$$

For a specific prison facility in a larger system, the conditions $f_{E}=0 ~\mathrm{and}~ f_{I}=0,$ can be achieved by admitting only susceptible inmates, while those carrying active TB are housed in facilities elsewhere. More generally, the condition is met if the ambient population is infection-free.

The *basic reproduction number*, denoted by $R_{0}$, of a disease in a population is defined as the average number of secondary infections that are produced when one infectious individual is introduced into a group of susceptible individuals. For more information see the books [2] of Anderson and May or [1] of Allen. For the model of [4], $R_{0}$ is given by the formula:

$$\begin{aligned} R_{0}=\frac{kc_{1}A}{\mu _{1}\mu _{2}}, \ \ \ \mathrm{with} \ \mu _{1}=\mu +r_{1}+k \ \mathrm{and} \ \mu _{2}=\mu +r_{2}+d. \end{aligned}$$

The basic reproduction number is a good indicator as to whether or not a disease will stay endemic in a population. If $R_{0}>1$ then each infectious individual produces, on average, more than one new infection, and the disease will persist in the population. If $R_{0}<1$, then on average an infected individual produces less than one new infected individual over the course of its infectious period, and it is more difficult for the infection to grow. In order to ensure that such a disease vanishes from the population, it may be necessary to impose conditions stronger than $R_{0}<1$. This problem is addressed in Theorem 1 below.

If the disease free equilibrium is *globally asymptotically stable*, it means that starting from any given state, in the long run the disease will vanish from the population. We now investigate for global stability of the disease free equilibrium of system (1) (subject to no inflow of infecteds) by using the Lyapunov function approach and we introduce the following invariant. Let

$$\begin{aligned} c_{*}=\max \left\{ c_{1}, ~ c_2, ~ c_{3}\left( \frac{\mu _{1}}{k}-1\right) \right\} . \end{aligned}$$

and let

$$\begin{aligned} R_{*}=\frac{kc_{*}A}{\mu _{1}\mu _{2}}. \end{aligned}$$

#### **Theorem 1**

*In model * (1) *let us consider the special case*, $f_E=0=f_I$. *If* $R_{*}<1,$ *then the disease free equilibrium* $P_{0}$ *is globally asymptotically stable.*

#### *Proof*

Starting with the condition $R_{*}<1$ we can choose numbers $\epsilon _{1}$ such that the following conditions are satisfied:

2 $$\begin{aligned} \left( \frac{k}{\mu _{1}}+\epsilon _{1}\right) c_{*}A-\mu _{2}<0, \end{aligned}$$

and let $a_{3}=\frac{k}{\mu _{1}}+\epsilon _{1}.$ Now choose $a_{2}>0$ such as to satisfy the following two inequalities:

3 $$\begin{aligned} a_{3}c_{*}A-\mu _{2}+a_{2}r_{2}<0 ~~~\mathrm{and} ~~~a_{2}r_{1}-\epsilon _{1}\mu _{1}<0. \end{aligned}$$

Next we choose $a_{1}$ sufficiently small such that

$$\begin{aligned} a_{1}c_{1}A+a_{3}c_{*}A-\mu _{2}+a_{2}r_{2}<0. \end{aligned}$$

We now define a function *V*(*S*, *E*, *I*, *T*) as follows,

4 $$\begin{aligned} V= \, & {} a_{1}(A-S)+a_{2}T+a_{3}E+I . \end{aligned}$$

Then it can routinely be shown that the function *V*(*S*, *E*, *I*, *T*) is Lyapunov at the disease-free equilibrium point $P_0$, and therefore $P_{0}$ is globally asymptotically stable. $\square$

Thus, if the system does not satisfy the condition $R_{*}<1$ for global stability, then as far as possible the authorities must intervene and make changes that will alter the values of the parameters so as to achieve this condition.

### Calibrating the model

As can be seen from the disease-free equilibrium, the number *A* turns out to be the maximum value of the varying population size *N*(*t*). For the case of the South African prison system, from the report [14] we deduce the value

$$\begin{aligned} A=160,000. \end{aligned}$$

In a disease model on general populations, the removal rate is calculated as the inverse of the life expectancy [3, 5, 9]. In 2015, life expectancy in South Africa was given as 67 years (y) [11], so the mortality rate for the general population would be $\frac{1}{67}\,\text{year}^{-1}$. In a prison model however, removal of individuals from the prison population entails both removal through death and removal by release from prison (assuming that the rate of escaping from prison is negligible). We proceed with determining this parameter. Henceforth, we assume *time* to be measured in *years*, y.

#### Numerical values for the removal rates

The removal rate $\mu$ and the additional removal rate *d* due to TB are calculated as below. Firstly we note that since we are working with probabilities, we can express $\mu$ as follows.

$$\begin{aligned} \mu =\mu _{p}+\mu _{m}-\mu _{m}\mu _{p}, \end{aligned}$$

where $\mu _{p}$ is the rate of release from prison and $\mu _{m}$ is the mortality rate in the prison, excluding death specifically as a result of TB. Deaths due to TB constitute a separate parameter.

*Release from prison* For calculating $\mu _{p}$ we used data from the public health paper [8]. The time served by prisoners is given in a frequency table which is convenient for calculating the average time served by inmates. We consider the awaiting trial detainees to stay for a nominal average period of 6 weeks, and the sentenced prisoners to serve on average $75\%$ of their sentence time. The value $\mu _{p}$ calculated in this way is

$$\begin{aligned} \mu _{p}=0.1789391 \ (\mathrm{year}^{-1}). \end{aligned}$$

*Mortality* In the classes *S*, *E* and *T* the probability of an individual being removed from the class due to death (except death as a result of TB) is denoted by $\mu _{m}$. For the class *I*, mortality due to TB-disease amplifies the removal rate by an additional increment *d*, which will be referred to as the disease-induced mortality rate. An estimate of $\mu _{m}$ can be obtained as follows. Consider a period of length $\tau$, over which the average value of the sum of the class sizes *S*, *E*, and *T* is denoted by *Q*. If the total number of deaths in these three classes during this period is *D*, then a value for $\mu _m$ can be estimated by the formula

$$\begin{aligned} \mu _m = \frac{D}{\tau Q} . \end{aligned}$$

The constant *d* can be estimated as follows. Consider a period of length $\tau _1$. If the total number of mortalities in the *I*-class during this period is $D_1$, then we estimate a value for *d* by the formula

$$\begin{aligned} \mu _{m}+d = \frac{D_1}{\tau _1 I}, \ \ \mathrm{i.e.,} \ \ d=\frac{D_1}{\tau _1 I} - \mu _{m} \ . \end{aligned}$$

For the years 2012–2015 the mortality rate is estimated using results from:

i. [14, Figure 7 on p. 42] for the numbers of inmates in total in SA prisons,
ii. [14, Figure 16 on p. 85] for the number of unnatural deaths,
iii. [14, Figure 20 on p. 91] for the number of natural deaths, and
iv. [21], the latter being particularly helpful in establishing an upper limit (20% of *A*) for the value of *S*(*t*).

The report [14] does not give the details of deaths in prison due to TB. In [14, Table 22 p. 71] TB comes up as the most prominent cause of natural death in prisons. Let us denote the rate of deaths due to TB by $\mu _{TB}$. According to the report in [15] we can take $\mu _{TB}=\frac{11}{80}\mu _{m}$ so that we can calculate $d=\mu _{TB}(1-\mu ).$

Our calculations yield the following values:

$$\begin{aligned} \mu _{m}=0.003628, ~~\mu _{TB}=0.02292, \mathrm{~~and}~~ \mu =0.18192 \ (\mathrm{year}^{-1}). \end{aligned}$$

Now note that *d* is the additional rate of removal due to TB. Thus

$$\begin{aligned} d=\mu _{TB}(1-\mu )=0.01876 \ (\mathrm{year}^{-1}). \end{aligned}$$

#### The parameters $c_i$

The formula [4], formula (17)] in the paper of Buonomo and Lacitignola stresses the fact that the force of infection is proportional to the population density. This means that when moving from a free population to a concentration camp, the force of infection will multiply by a significant factor, and in a prison population it will be another factor higher.

Using 2015 data obtained from [21] and life expectancy as from [11], a simple calculation shows that a lower bound for the effective contact rate (let us denote it by $c_0$) for TB in South Africa (the entire population) yields (a lower bound)

$$\begin{aligned} c_0=\frac{(55-44) \mathrm{\ million}}{67\times 390,000\times (44 \mathrm{\ million})}=1.5308\times 10^{-7} \mathrm{year^{-1}}. \end{aligned}$$

When applying this to a subpopulation, this parameter should be scaled up, inversely to the change in population size. Furthermore, in the prison system we expect a value a few factors higher. In order not to present a situation worse than reality, for the prison system we use a figure $c_1 = 1.5\,c_0 \times \frac{P}{A}$ where *P* is the 2015 population size of South Africa. Thus we obtain a value

$$\begin{aligned} c_1 = 0.00007893 \ \mathrm{year}^{-1}. \end{aligned}$$

For the coefficient $c_3$ in comparison with the coefficient *k* (valuated in “Other parameters” section), since $c_3$ is multiplied by *E* we allow a nominal value

$$\begin{aligned} c_3=k/(2A). \end{aligned}$$

The treatment time is usually 6 months, see [6] for instance. This means that the rate of departure from the *T*-class per year is 2*T*. In the model the flow out of the *T*-class into the *E*-class is assumed to be proportional to *TI*. For this reason we choose a value of $c_2$ at

$$\begin{aligned} c_2=2(10/A), \end{aligned}$$

such that when *I* reaches a reasonably high value such as around $I=0.1 A$, then the average time spent in class *T* is approximately 6 months.

#### Other parameters

The progression rate from the exposed and infectious classes to treatment class are, $r_{1}=0.30$ and $r_{2}=0.5$ respectively [4]. Using 2015 data obtained from [21], for the transfer rate *k* in South Africa (the entire population) from *E* to *I* we obtain a value $450,000/[0.8\,(55 \text{million})]$. The value used in [4] (i.e., 0.1) is a factor 10 times higher than this rough calculation. For our purposes we use the value

$$\begin{aligned} k=0.05. \end{aligned}$$

In a prison system where high quality screening is performed, the parameters $f_{S},\,f_{E},\,\text{and}\, f_{I}$ can be determined fairly accurately. In the absence of such facilities, the best estimates for these parameters are to derive them from the proportions, in the bigger population, of susceptible, latent and infectious. Thus we have the following:

$$\begin{aligned} f_{S}=0.2,\quad ~ f_{E}=0.74, \quad \text{and}\quad f_{I}=0.06. \end{aligned}$$

#### Initial conditions for simulation

We require initial conditions in order to run simulations that can be useful for projection of numbers in the future. According to the annual report of the Department of Correctional Services [16] we know the numbers of infectious TB patients and those under treatment. Thus we know $I(t_{15}) \, \text{and}\, T(t_{15}),\, t_{15}$ denoting the time 31 March 2015. We also have a value for $N(t_{15})=159,563.$ In order to find a reasonable split of the number

$$\begin{aligned} N(t_{15})-[I(t_{15})+T(t_{15})] \end{aligned}$$

between $S(t_{15}) ~\mathrm{and}~ E(t_{15}),$ since $S+E+I+T=N,$ we recall from [21] that approximately only 20% of the South African population are susceptible, i.e., has never been infected with the TB bacterium. These observations lead to the following initial condition:

$$\begin{aligned} S(t_{15})=32,000,\quad E(t_{15})=107,000,\quad I(t_{15})=3500, \quad T(t_{15})=17,100. \end{aligned}$$

### Simulations

Through simulations we utilize the model to investigate the effect of interventions, by making future projections of the levels of TB prevalence in a prison system. We test the various scenarios, including the case of no inflow of infecteds. Model system (1) has been evaluated for global stability in Theorem 1, which has assured us that if the condition of the theorem is satisfied, then starting from any point in our model system (1), the disease will ultimately vanish from the prison population. We will also illustrate this result by means of simulation.

## Results and discussion

We presented and motivated a model for the population dynamics of TB in a prison or prison system. Parameter values for the South African prison population have been calculated from data in the open literature and these are summarized in Tables 1 and 2. We proved a theorem, Theorem 1, describing conditions that will guarantee the ultimate eradication of TB for a prison system. Sample simulations have been run, to be discussed below.

**Table 1:** Model parameters and initial conditions

Parameter	Numerical value	Sources
$\mu$	0.18192	[8, 14]
*d*	0.01876	[14, 15]
$r_{1}$	0.30	[4]
$r_{2}$	0.50	[4]
*k*	0.05	[4, 21]
*A*	1,600,000	[14]
$S_{t_{15}}$	32,000	[14]
$E_{t_{15}}$	107,000	[14]
$I_{t_{15}}$	3500	[14]
$S_{t_{15}}$	17,100	[14]

### No inflow of infectives

We first provide an analysis of our model system without the inflow of the infectives, i.e., when $f_{E}=0 ~\mathrm{and}~ f_{I}=0.$ In this case we use the parameters from Table 1, while varying the values of the parameters $c_i$ not listed in Table 1. The reason for varying these parameters is to be able to obtain different values of $R_*$ to illustrate Theorem 1.

**Fig. 1:**
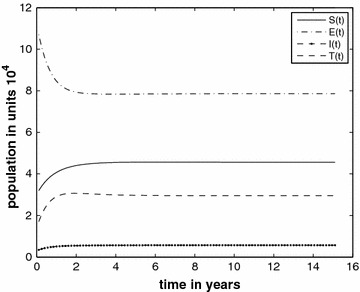
Prison population in different classes without the inflow of infectives and $R_{*}=1.72$

Figure 1 (Case 1) shows the trend of all classes over 15 years with $c_{1}=0.00008$ and we compute $R_{*}=1.72$. The graphs indicate that the disease will persist in the prison population.

**Fig. 2:**
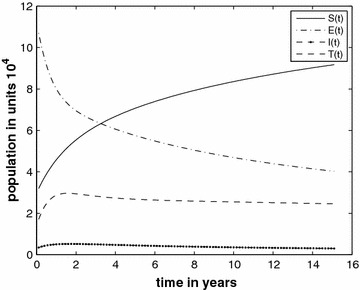
The prison population in different classes without the inflow of infectives and $R_{*}=0.86$

Figure 2 (Case 2) shows variation of susceptible, exposed, infected and treated classes over 15 years, with $c_{1}=0.00004$ and in this case we obtain $R_{*}=0.86$. Under these conditions the theorem assures us that the TB disease will vanish.

**Fig. 3:**
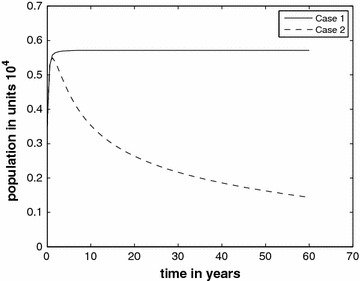
Infective class without the inflow of infectives for two cases $R_{*}=1.72$ and $R_{*}=0.86$

In Fig. 3, we show the infectious classes *I* of both Case 1 and Case 2 for comparison, and we stretch the time horizon to 60 years. For Case 2 the graph gives an indication of how fast the infectious class falls to zero. In order to make it vanish faster, further intervention is necessary to reduce the value of $R_*$.

**Table 2:** Inflow and contact rates

Parameter	Numerical value	Sources
$c_{1}$	0.00007893	[21]
$c_{2}$	20/*A*	[6]
$c_{3}$	*k*/(2*A*)	Estimated
$f_{S},\,f_{E},\,f_{I}$	0.2, 0.74, 0.06, respectively	[14]

### General case

**Fig. 4:**
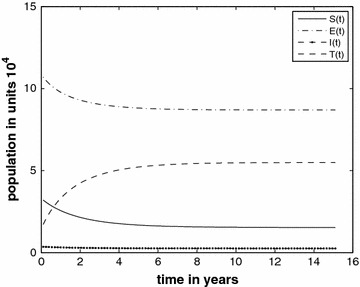
The different classes with the inflow of infectives at $f_E=0.74$ and $f_I=0.06$ (Case A)

**Fig. 5:**
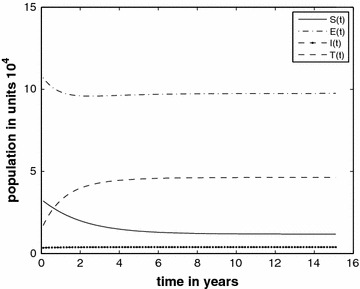
The different classes with reduced inflow of infectives $f_E=0.77$, $f_I=0.03$ (Case B)

**Fig. 6:**
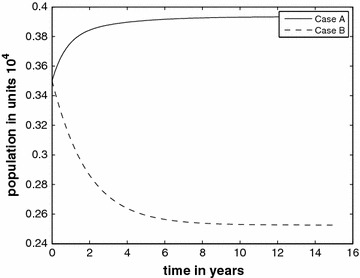
Comparison of Infective classes with $f_I=0.06$ (Case A) and $f_I=0.03$ (Case B)

In this section, we will consider the general case, i.e., the model with the inflow of the infective into the prison population. We use the paremeters from Tables 1 and 2. We consider two inflow scenarios for comparison. We first consider the case (call it Case A) with $f_E=0.64$ and $f_I=0.06$. The curves are depicted in Fig. 4. In Fig. 5 (Case B) we use the inflow parameters at the values $f_E=0.77$ and $f_I=0.03$. The comparison shows the effect of reduction of inflow of infectious individuals. In order to better compare visually, the *I*-classes of Case A and Case B are drawn on the same system of axes in Fig. 6. We see a remarkable drop in the *I* numbers when the inflow of infecteds is halved.

These graphs demonstrate the extent to which this model can be utilized when planning to roll out an intervention strategy.

## Conclusions

We started with an existing population model of TB that was applied to a specific crowded environment (concentration camps). This model was adjusted to apply to prisons or prison systems. In this compartmental model we allowed for inflow of infectives into classes other than just the susceptible class. In fact, such inflow has to be accommodated in the model if there is TB infection in the ambient population. On the removal side it is important to note that release from prison is the main component, complemented by removal through death. We have described conditions (for mathematical stability of the disease free state of the system) that will cause the TB infection to vanish from the prison population. It was observed that if at a specific prison site or system there is no inflow of infected individuals, then the disease will vanish from the prison provided that the numerical value of the invariant $R_*$ is below unity.

For the case of the South African prison system, most of the crucial parameters of the model were calculated using data from public domain prison data. Other parameters, including initial conditions for computations, were obtained from data in various published literature, together with interpolation methods. As illustrated in the previous section, the model can be utilized in making future projections of the levels of prevalence of TB, and to quantify the effect of interventions such as screening, treatment or reduction of transmission parameter values through improved living conditions for the inmates.

## Abbreviations

SEIT: susceptible-latent-infectious-treatment
TB: tuberculosis
WHO: World Health Organization
